# Study of Mental Health Status of the Resident Physicians in China During the COVID-19 Pandemic

**DOI:** 10.3389/fpsyg.2022.764638

**Published:** 2022-03-15

**Authors:** Shuang-Zhen Jia, Yu-Zhen Zhao, Jia-Qi Liu, Xu Guo, Mo-Xian Chen, Shao-Ming Zhou, Jian-Li Zhou

**Affiliations:** ^1^Division of Gastroenterology, Shenzhen Children’s Hospital, Shenzhen, China; ^2^Co-Innovation Center for Sustainable Forestry in Southern China and Key Laboratory of National Forestry and Grassland Administration on Subtropical Forest Biodiversity Conservation, College of Biology and the Environment, Nanjing Forestry University, Nanjing, China

**Keywords:** 2019 novel coronavirus, Chinese resident physicians, mental health, SCL-90 symptom self-rating scale, investation

## Abstract

**Objective:**

Investigating the mental health status of Chinese resident physicians during the 2019 new coronavirus outbreak.

**Methods:**

A cluster sampling method was adopted to collect all China-wide resident physicians during the epidemic period as the research subjects. The Symptom Checklist-90 self-rating scale was used to assess mental health using WeChat electronic questionnaires.

**Results:**

In total, 511 electronic questionnaires were recovered, all of which were valid. The negative psychological detection rate was 93.9% (480/511). Among the symptoms on the self-rating scale, more than half of the Chinese resident physicians had mild to moderate symptoms of mental unhealthiness, and a few had asymptomatic or severe unhealthy mental states. In particular, the detection rate of abnormality was 88.3% (451/511), obsessive-compulsive symptoms was 90.4% (462/511), the sensitive interpersonal relationship was 90.6% (463/511), depression abnormality was 90.8% (464)/511), anxiety abnormality was 88.3% (451/511), hostility abnormality was 85.3% (436/511), terror abnormality was 84.9% (434/511), paranoia abnormality was 86.9% (444/511), psychotic abnormalities was 89.0% (455/511), and abnormal sleeping and eating status was 90.8% (464/511). The scores of various psychological symptoms of pediatric resident physicians were significantly lower than those of non-pediatrics (*p* < 0.05).

**Conclusion:**

The new coronavirus epidemic has a greater impact on the mental health of Chinese resident physicians.

## Introduction

The novel coronavirus (2019-nCoV) infection broke out in China in November 2019 and quickly developed into a global pandemic (Zhou et al., 2020). As of July 26, 2020, more than 15 million cases of COVID-19 have been documented worldwide, with nearly 618,000 deaths (Atzrodt et al., 2020; Wu and McGoogan, 2020). This has become a public health emergency of international concern and will surely affect everyone in the world. In the SARS outbreak 17 years ago, some researchers observed adverse psychological problems in medical staff (Chan and Huak, 2004; Chen et al., 2005; Phua, 2005; Lin et al., 2007; Wu et al., 2009).

The new coronavirus is highly contagious and can be transmitted through multiple channels, such as respiratory droplets, air, and direct contact. It has become a global epidemic in a short period of time (Rothan and Byrareddy, 2020; Wu and McGoogan, 2020). The human population is generally susceptible and lacks specific medicines to target this virus, thus resulting in a relatively high mortality rate (Tebala and Bond-Smith, 2020). In the early stages of the disease outbreak, people generally lacked awareness of the new coronavirus, and the implementation of prevention and control measures, such as lockdowns, definitely had a huge impact on the quality of life and physical and mental health of the human population (Wu and McGoogan, 2020).

In the past 20 years, there have been many global epidemics, including the severe acute respiratory syndrome (SARS) that was prevalent in China and Asian countries in 2003, the H1N1 influenza that broke out in 2009, the Middle East Respiratory Syndrome (MERS) that occurred in 2012, and the Ebola virus pandemic that occurred in Africa in 2014 (Peiris et al., 2003; Baden et al., 2009; Zumla et al., 2015; Leligdowicz et al., 2016). Each of these had different effects on different regions and different groups of people. Among them, medical personnel are the frontliners in the fight against these deadly viruses. During the SARS period, significant psychological changes were found in patients with SARS and high-risk medical staff, suggesting that they may have varying degrees of negative psychological states (Kisely et al., 2020). Young resident physicians who were directly involved in the diagnosis and treatment of patients with new coronavirus infections had mental health and psychological risks that deserve more attention. Therefore, the purpose of this study is to assess the mental health status of Chinese resident physicians using the Symptom Checklist-90 (SCL-90) self-rating scale and to understand the impact of the epidemic on the mental health of these residents.

## Materials and Methods

### Material

From November 2019 to May 2020, the resident physicians who were undergoing residency training process in China, including 3 classes, namely, 2017, 2018, and 2019, were included as research subjects. These resident physicians worked in five major departments, including internal medicine, surgery, obstetrics and gynecology, pediatrics, and emergency/ICU. Resident physicians from the departments of psychology, oral cavity, ENT, ophthalmology, rehabilitation, etc., were not included in the research.

### Methods Used in This Study

The survey, conducted in the form of an answer sheet, including the survey content, purpose, filling method, and requirements for reporting time, was sent out by WeChat or QR-code. Researchers conducted a comprehensive analysis and evaluation of the submitted questionnaire later on. The questionnaire included general information about these resident physicians, such as gender, age, educational background, grade of training, departments, whether they were on duty during the epidemic, etc. The SCL-90 self-rating scale consisted of 90 items divided into 10 major factors, namely, somatization, obsessive-compulsive symptoms, horror, hostility, anxiety, depression, paranoia, interpersonal sensitivity, mental illness, and eating and sleeping habits. The total score was 450 points, and a score of over 160 indicated positive symptoms. Furthermore, each factor score (S) was divided into four levels, that is, S ≤ 1 was regarded as asymptomatic, 2 points ≤ S < 3 was regarded as mild symptoms, 3 points ≤ S < 4 was considered moderate symptoms, and S ≥ 4 was considered severe symptoms. This study was reviewed by the hospital ethics committee, and all research subjects provided informed consent.

### Statistical Methods

Descriptive analysis was used for general information. All the measurement data passed the normal distribution test and the homogeneity of variance test. Data with the normal distribution and had homogeneous variance were analyzed by *t*-test or variance analysis and were labeled as X ± SD. The data that did not fit the normal distribution or were with uneven variance were subjected to the non-parametric test rank sum test. The results were described in quartiles Q1–Q3. Quantity data passed the Chi-square or Fisher test. Scores of psychological symptoms were compared using the Kruskal–Wallis test in all departments and the Mann–Whitney *U*-test between any two departments. A two-sided test at α = 0.05, *p* < 0.05 indicated that the differences were statistically significant.

All statistical analyses were performed using SPSS 26.0 software and R 3.5.1 for Windows (R Foundation for Statistical Computing, Vienna, Austria) with the “ggplot2” and “ggpubr” packages.

## Results

### Sample

A cluster sampling method was adopted to investigate a total of 511 resident physicians who were undergoing residency training in China during the epidemic. Among them, 297 were males and 214 were females, accounting for 58.1 and 41.9%, respectively. The enrolled resident physicians were 20–29 years old, with an average age of 23.9 ± 1.85 years old. In particular, a total of 197 (38.6%) of these resident physicians were from the class of 2017, 239 (46.8%) from the class of 2018, and 75 (14.7%) from the class of 2019. Furthermore, 239 (46.8%) held bachelor’s degrees, and 255 (49.9%) and 17 (3.3%) were masters and doctors, respectively. A total of 287 (56.2%) and 224 (43.8%) people were on and off duty, respectively.

### SCL-90 Scale Test Results and Severity Analysis on 511 Resident Physicians

Among 511 resident physicians, 480 (93.9%) had a total score above the normal range on the SCL-90 test. In particular, the detection rate of somatization abnormalities was 88.3% (451/511), the detection rate of obsessive-compulsive symptoms was 90.4% (462/511), the detection rate of interpersonal sensitivity abnormalities is 90.6% (463/511), the detection rate of depression abnormalities was 90.8% (464/511), the detection rate of anxiety abnormality was 88.3% (451/511), the detection rate of hostile abnormality was 85.3% (436/511), the detection rate of terror abnormality was 84.9% (434/511), the abnormal detection rate of paranoia was 86.9% (444/511), the detection rate of psychotic abnormalities was 89.0% (455/511), and the detection rate of sleeping and eating abnormalities was 90.8% (464/511).

Further comparison of the degree of various symptoms and mild abnormalities accounts for the highest proportion among tested resident physicians. For example, somatization accounted for 47.4% (242/511), compulsion for 47.2% (241/511), interpersonal relationship for 42.9% (219/511), depression for 46.2% (236/511), anxiety for 46.6% (238/511), hostility for 35.4% (181/511), terror for 44.0% (225/511), paranoia for 44.6% (228/511), psychosis for 50.1% (256/511), and sleeping and eating for 46.0% (235/511). Among various symptoms, severe abnormalities accounted for the least proportion of tested doctors, with a range of 12.5–23.9% for every tested parameter, such as somatization for 17.2% (88/511), compulsion for 15.8% (81/511), interpersonal relationships for 12.9% (66/511), depression for 12.9% (66/511), anxiety for 12.5% (64/511), hostility for 23.9% (122/511), horror for 15.7% (80/511), paranoia for 12.9% (66/511), psychotic for 16.0% (82/511), and sleeping state for 13.9% (71/511).

### Comparison of Psychological Evaluation Scores of the Resident Physicians in Different Specialties

Group comparisons suggested that the scores of 10 psychological symptoms of resident physicians in three departments, including internal medicine, surgery, and emergency/ICU, were significantly higher than those of pediatric resident physicians (*p* < 0.05). In contrast, there is no significant difference in the scores of these three departments themselves (*p* > 0.05). Furthermore, resident physicians from obstetrics and gynecology presented higher scores on two parameters, such as somatization and compulsive psychological symptoms, than the scores of pediatric resident physicians (*p* < 0.05), whereas no differences were observed in the rest of the eight psychological symptoms evaluated in this study (*p* > 0.05). We found that resident physicians in the emergency or ICU departments have more serious psychological problems than resident physicians in pediatric departments, including abnormalities in somatization, depression, and eating and sleeping habits (Table 1).

**TABLE 1:** Scores of psychological symptoms among resident physicians in different specialties.

Items	Department					*P*
	Medical	Surgical	Obstetrics and gynecology	Pediatrics	Emergency room/ICU	
Somatization	2.87 ± 0.78	2.99 ± 0.81	2.98 ± 0.73	2.50 ± 1.15	3.02 ± 0.85	0.010
Compulsive symptom	2.93 ± 0.77	3.02 ± 0.83	2.98 ± 0.71	2.57 ± 1.06	3.07 ± 0.74	0.020
Interpersonal sensitivity	2.98 ± 0.74	3.04 ± 0.78	2.94 ± 0.75	2.58 ± 1.09	3.08 ± 0.68	0.035
Depression	3.00 ± 0.71	3.02 ± 0.80	2.96 ± 0.69	2.52 ± 1.05	3.05 ± 0.77	0.008
Anxiety	2.85 ± 0.72	2.96 ± 0.82	2.77 ± 0.82	2.45 ± 1.04	3.00 ± 0.75	0.004
Hostility	3.11 ± 0.90	3.05 ± 0.95	2.76 ± 0.84	2.59 ± 1.19	3.07 ± 1.01	0.006
Phobia	2.88 ± 0.80	2.96 ± 0.89	2.70 ± 0.85	2.44 ± 1.13	2.95 ± 0.90	0.001
Paranoid	2.88 ± 0.81	2.93 ± 0.83	2.73 ± 0.76	2.45 ± 1.10	3.04 ± 0.71	0.002
Psychotic symptom	2.93 ± 0.74	2.94 ± 0.82	2.81 ± 0.74	2.46 ± 1.10	3.01 ± 0.84	0.005
Sleeping and eating habits	2.89 ± 0.74	3.01 ± 0.80	2.83 ± 0.81	2.53 ± 1.12	3.13 ± 0.73	0.002

**FIGURE 1:**
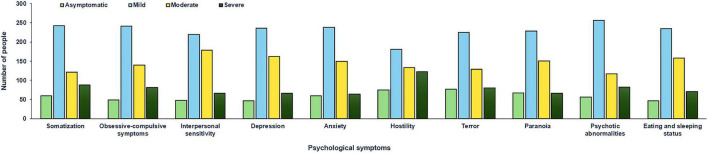
Distribution characteristics of psychological symptoms among 511 resident physicians.

**FIGURE 2:**
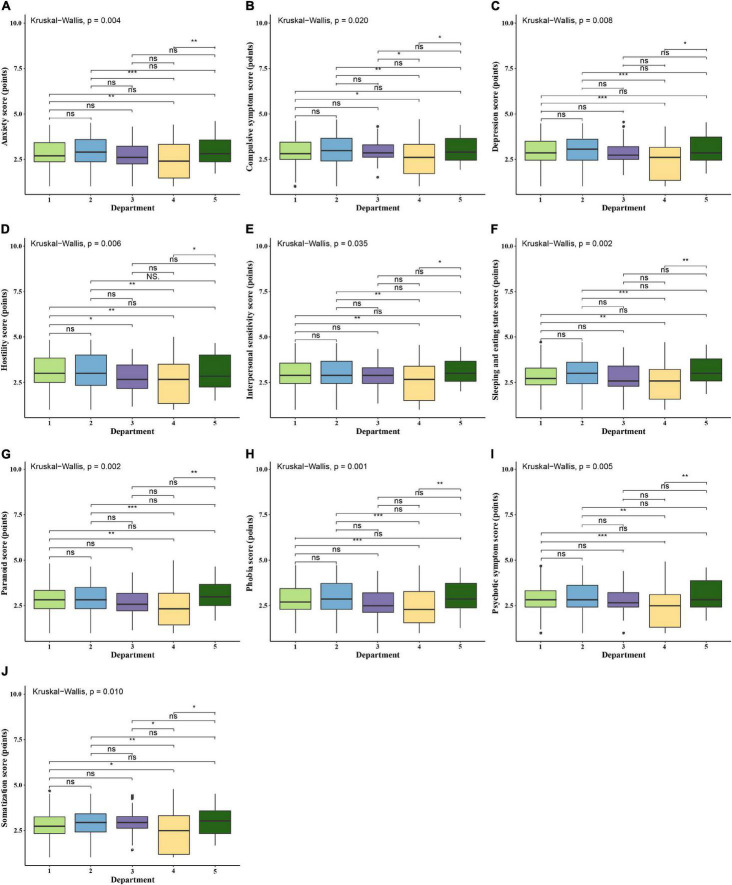
Comparison of psychological evaluation scores of the resident physicians in different specialties. ns, not statistically significant. *p < 0.05; **p < 0.01; ***p < 0.001; ****p < 0.0001. 1, Medical department; 2, Surgical department; 3, Obstetrics and gynecology department; 4, Pediatrics department; 5, Emergency room/ICU. **(A)** Comparison of scores of “Anxiety” among resident physicians in different specialties. **(B)** Comparison of scores of “Compulsive symptom” among resident physicians in different specialties. **(C)** Comparison of scores of “Depression” among resident physicians in different specialties. **(D)** Comparison of scores of “Hostility” among resident physicians in different specialties. **(E)** Comparison of scores of “Interpersonal sensitivity” among resident physicians in different specialties. **(F)** Comparison of scores of “Sleeping and eating habits” among resident physicians in different specialties. **(G)** Comparison of scores of “Paranoid” among resident physicians in different specialties. **(H)** Comparison of scores of “Phobia” among resident physicians in different specialties. **(I)** Comparison of scores of “Psychotic symptom” among resident physicians in different specialties. **(J)** Comparison of scores of “Somatization” among resident physicians in different specialties.

Among the 511 resident physicians, 83 (16.2%) were pediatric resident physicians, and 428 (83.8%) were non-pediatric resident physicians (e.g., internal medicine, surgery, obstetrics and gynecology, and emergency/ICU). Through independent sample *t*-test analysis, it was shown that the scores of various psychological symptoms of doctors from the pediatric department were significantly lower than the scores of resident physicians with non-pediatric specialties (*p* < 0.05). The positive detection rate of residents is relatively high, which is mainly manifested as a mild psychological disorder (Figure 1). In particular, some resident physicians have moderate psychological problems and very few resident physicians have severe psychological conditions (Figure 2).

## Discussion

Up to February 11, 2020, a total of 72,314 COVID-19 cases have been reported in mainland China. Most of these patients were aged between 30 and 79 years old, accounting for 86.6% of the total reported cases (Chinese Center For Disease Control And Prevention, 2020). Relatively few reports of children and adolescents were documented. In recent years, with the continuous increase of public emergencies, the demand for high-quality emergency care is increasing. In fact, in our traditional society, people, as members of society, will have varying degrees of psychological impact due to the acceleration of their lives, changes in family conflicts, and pressure at work. In 2018, Yu et al. (2019) used a cluster sampling method in 12 cities in China and applied a large-scale SCL-90 test to survey Chinese people whose age ranged from 18 to 60 years. This study found that young people may have higher risk of having psychological problems. Furthermore, a British survey also suggested that 31.6% of doctors felt excessive anxiety, 61.7% felt depression, and 46.7% had sleep disorders (Vincent et al., 2015). It shows that the psychological changes in medical staff fluctuate with negative stimulus factors, i.e., pain, anxiety, illness, and death, they face each day of their lives.

As a special group in clinical work, resident physicians have relatively little clinical experience and lack of emergency response. Thus, their psychological aspects need to withstand greater challenges. Current studies have shown that residents are prone to psychological problems. In addition, the incidence of depression or depressive symptoms among them is annually increasing from 20.9 to 43.2% (Al-Maddah et al., 2015; Mata et al., 2015). At the same time, they appear to be a high-risk group for suicide. This demonstrated that the new coronavirus pandemic has a greater psychological impact on young doctors. As clinical medical staff at the frontline, they initially had little understanding of the new coronavirus and, therefore, had insufficient psychological preparation and knowledge. Furthermore, they are exposed to medical sharps, blood, and body fluids of patients, increasing the risk of infection. Moreover, their work environment has changed due to the increase in workload and the need for prevention and control measures, which, along with the fear of infections for themselves and their family members, has greatly increased their risk for psychological problems. If these psychological problems are not immediately solved, they will not only endanger the physical and mental health of fellow doctors, but also cause medical errors and mistakes during clinical treatment (Zhang et al., 2019). Therefore, as an important stage in the transformation of qualified doctors, the mental health of young doctors should be carefully monitored. Long-term mental illness will lead to severe diseases in the neurological or endocrine systems (Deng et al., 2018). Therefore, resident physicians have become a high-risk group for mental illnesses. Hence, their psychological problems should receive extensive attention.

A psychological study of 330 resident physicians found that the overall burnout rate in China before COVID-19 was 71.4% (Huang et al., 2020). In our study, we had a higher percentage of psychological problems among resident physicians during the COVID-19 pandemic. Judging from the positive detection rate of psychological symptoms, psychological problems such as hostility, interpersonal sensitivity, depression, and compulsion were major causes. In particular, the severe unhealthy mental state was mainly caused by two symptoms, namely, hostility and somatization. Hostility is manifested as boredom in daily life and an inability to control one’s temper, whereas somatization is mainly manifested in physical discomfort from time to time, including discomforts in the cardiovascular, gastrointestinal, and respiratory systems, such as headaches, backaches, and muscle aches. Recent studies have shown that after an emergency breakout, approximately 70% of psychological trauma can be healed automatically, whereas the remaining 30% of these cases will later have a series of psychological and physical symptoms, including anxiety, depression, physical disorders, eating and sleep disorders, and alcohol or drug dependence (Yao, 2008). Therefore, it is beneficial for public health to pay special attention to the mental health of resident physicians. Building a good psychological support and intervention system and assisting these frontline medical staff to actively cope with pressure are conducive approaches, stimulating the enthusiasm of these young doctors (Xu et al., 2018).

There are high requirements in the comprehensive quality of medical staff in order to accommodate the characteristics of severe patient conditions, frequent emergencies, and heavy workload in emergency departments. Therefore, it not only requires strong medical technology to deal with critically ill patients, but also strong physical strength to cope with the heavy workload. At the same time, doctors in emergency departments require strong psychological abilities to deal with emergencies such as their own inability, the suffering and helplessness of patients, family sadness, and medical disputes. These matters will lead to the medical illness of doctors in emergency departments (Zhang et al., 2018). In this study, we found that resident physicians in emergency/ICU departments have more serious psychological problems than resident physicians in pediatric departments. These problems include abnormalities in somatization, depression, and eating and sleeping habits, demonstrating that resident physicians in emergency/ICU departments are prone to psychological problems. Furthermore, it is common for resident physicians to experience frequent discomfort in their cardiovascular, gastrointestinal, and respiratory systems, along with headaches, backaches, muscle aches, and other symptoms in their daily lives. In addition, they are suffering from disappointment, pessimism, and suicidal ideas due to a lack of motivation in their daily lives.

The emergency and ICU departments have become the first places for patients, making residents face a greater degree of high pressure. Therefore, it is very important to improve the psychological quality of emergency care and reduce the negative psychological impacts caused by patients’ deaths, especially for resident physicians who have just entered emergency care. In the continuing education of modern medicine, the training institutions and teachers should focus on training the professional level of the young doctors, improving their communication skills and their ability to respond to emergencies. Furthermore, we should continuously improve the psychology of the emergency/ICU departments through regular psychological consultation for resident physicians, further strengthening their psychological endurance to negative impacts in order to achieve better performance in clinical work. In addition, the government needs to publicize efforts that have been made by hospitals and doctors to ease the doctor–patient relationship by ensuring that the public has a positive awareness of doctors, especially in emergency and ICU departments (Shen et al., 2017).

## Conclusion

In conclusion, according to the survey results of this study, the mental health status of 511 resident physicians was lower than the national standard performance during the COVID-19 epidemic period. Factors such as whether these young doctors were on duty or not and various performances among different departments have suggested that we should carry out targeted psychological interventions in our daily work. In addition, particular attention needs to be paid to doctors in emergency and ICU departments who work during the epidemic. However, the shortcomings of this survey include our method of distribution of the questionnaires. We distributed electronic questionnaires using the internet, hence it is not yet possible to accurately verify the identity of the research subjects and the authenticity and representativeness of the content filled in them. Furthermore, the research sample size is not yet sufficient, and there is a lack of data tracking mechanisms, so it is temporarily impossible to assess whether the above psychological problems will exist for a long time. In the future, it is urgent to carry out a study with a larger sample size and longer follow-up observations to better understand the impact of the COVID-19 epidemic on the mental health of Chinese resident physicians.

## Data Availability Statement

The original contributions presented in the study are included in the article/supplementary material, further inquiries can be directed to the corresponding author/s.

## Ethics Statement

Ethical review and approval was not required for the study on human participants in accordance with the local legislation and institutional requirements. Written informed consent for participation was not required for this study in accordance with the national legislation and the institutional requirements.

## Author Contributions

J-LZ and S-MZ designed the research. J-LZ, S-MZ, and S-ZJ conducted the research. S-ZJ analyzed the data, wrote the manuscript, and was primary responsibility for final content. J-LZ, S-MZ, M-XC, S-ZJ, Y-ZZ, J-QL, and XG critically read and revised the manuscript. All authors read and approved the final manuscript.

## Conflict of Interest

The authors declare that the research was conducted in the absence of any commercial or financial relationships that could be construed as a potential conflict of interest.

## Publisher’s Note

All claims expressed in this article are solely those of the authors and do not necessarily represent those of their affiliated organizations, or those of the publisher, the editors and the reviewers. Any product that may be evaluated in this article, or claim that may be made by its manufacturer, is not guaranteed or endorsed by the publisher.
